# 306. Association of Antibiotic Use and Development Secondary Infection from *Clostridium difficile*, Multidrug-Resistant Bacteria, and *Candida* in Hospitalized Patients with History of COVID-19

**DOI:** 10.1093/ofid/ofab466.508

**Published:** 2021-12-04

**Authors:** Jessica K Costales, Helen Lee, Kathleen A Quan, Keith M Madey, Kurt McArthur, Shruti K Gohil, Susan S Huang, Donald Forthal, Steven Park

**Affiliations:** 1 Kaiser Permanente Los Angeles Medical Center, Los Angeles, CA; 2 UCI Health, Orange, California; 3 University of California Irvine Health, Orange, California; 4 University of California Irvine Medical Center, Lakewood, California; 5 UCI Irvine Medical Center, Santa Ana, California; 6 UC Irvine School of Medicine, IRVINE, California; 7 University of California, Irvine, Irvine, CA; 8 University of California, Irvine School of Medicine, Irvine, California

## Abstract

**Background:**

There is increasing evidence that patients hospitalized with COVID-19 receive unnecessary antibiotics. The consequences of antibiotic overuse as it relates to antimicrobial resistance and development of secondary infections remains uncertain. The objective of this study is to compare antibiotic prescription patterns in patients with a history of COVID-19 to those without a history of COVID-19 and determine if there are differences in the frequency of secondary infections from *Clostridioides difficile* (*C. difficile*), multidrug-resistant (MDR) bacteria, and *candida* infections.

**Methods:**

This study is a single-center, retrospective cohort study of 18,757 adults hospitalized during the COVID-19 pandemic from March 1, 2020 to March 31, 2021. Patients were stratified as COVID-19 positive, throughout all hospitalizations subsequent to the date of initial positivity, or COVID-19 negative. Differences in antibiotic practice patterns between the two groups were quantified using days of therapy per 1000 patient days (DOT/1000 PD). The frequency of *C. difficile* infection, MDR-bacteria, and *candida* infections were assessed among the two groups.

**Results:**

During the 12-month study period, on average, the COVID-19 positive group received 21.81% more antibiotics than COVID-19 negative patients, with up to 56.15% increase seen in the first month of the pandemic (Table 1, Figure 1) The COVID-19 positive group had an increased frequency of Candidemia (0.73% versus 0.18%, p< .00001) and decreased isolation of ESBL organisms (1.17% versus 1.87%, p< 0.01416) compared to the COVID-19 negative group. There were no significant differences in frequency of *C. difficile* infection, isolation of other MDR-organisms, or *Candida auris* between the two groups. (Table 2)

Table 1. Antibiotic days of therapy in COVID-19 positive and COVID-19 negative patients.

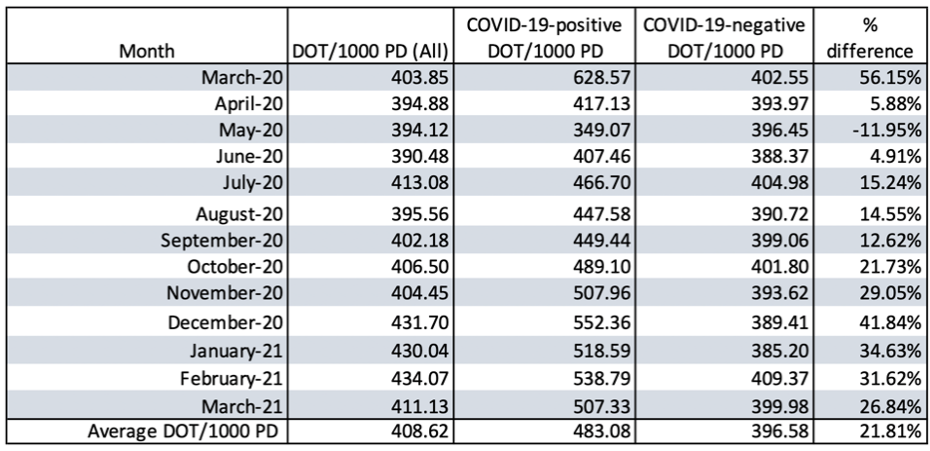

Figure 1. Antibiotic days of therapy in total cohort, COVID-19 positive, and COVID-19 negative patients.

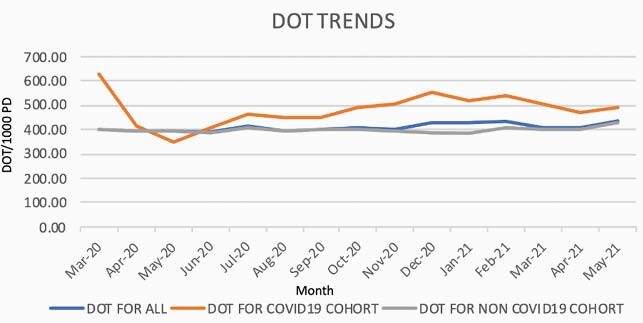

Table 2. Frequency of secondary infections in COVID-19 positive and COVID-19 negative patients

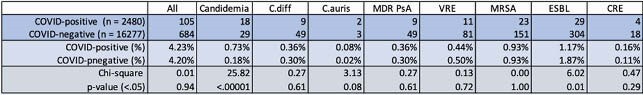

**Conclusion:**

Patients with a history of COVID-19 infection received an average of 21.81% more antibiotics, have higher rates of candidemia, but lower rates of ESBL infection than those without a history of COVID-19 infection. The potential increase in antibiotic exposure could account for the increase in candidemia in patients with a history of COVID-19. Future studies include investigating the decrease in ESBL infections seen, perhaps due to receipt of broad antibiotics in COVID-19 patients that target ESBL bacteria.

**Disclosures:**

**Shruti K. Gohil, MD, MPH**, **Medline** (Other Financial or Material Support, Co-Investigator in studies in which participating hospitals and nursing homes received contributed antiseptic and cleaning products)**Molnycke** (Other Financial or Material Support, Co-Investigator in studies in which participating hospitals and nursing homes received contributed antiseptic and cleaning products)**Stryker (Sage**) (Other Financial or Material Support, Co-Investigator in studies in which participating hospitals and nursing homes received contributed antiseptic and cleaning products) **Susan S. Huang, MD, MPH**, **Medline** (Other Financial or Material Support, Conducted studies in which participating hospitals and nursing homes received contributed antiseptic and cleaning products)**Molnlycke** (Other Financial or Material Support, Conducted studies in which participating hospitals and nursing homes received contributed antiseptic and cleaning products)**Stryker (Sage**) (Other Financial or Material Support, Conducted studies in which participating hospitals and nursing homes received contributed antiseptic and cleaning products)**Xttrium** (Other Financial or Material Support, Conducted studies in which participating hospitals and nursing homes received contributed antiseptic and cleaning products)

